# The Retort Courteous

**Published:** 1923-07

**Authors:** A. W. Fuller

**Affiliations:** 31 New Cavendish Street, W.1


### The Retort Courteous.

SIR,—Regarding a review, appearing in your June issue, on my book on Anæmia, the writer, in my opinion, over-steps the limits of just criticism. He reviews not so much the book as the author and his own deductions of what he supposes was in my mind when I wrote it. A reviewer of a book one would expect to have some literary attainments, but an elementary schoolboy could hardly express his meaning in a more illiterate fashion than your reviewer when he says "The science of medicine is team-work!" Team-work is hardly a science.

A. W. FULLER, M.D.

31 New Cavendish Street, W.1.

[Our correspondent appears to lack in courtesy at least as much as he believes our reviewer to lack in literary attainments.—EDITOR, THE HOSPITAL AND HEALTH REVIEW.]

